# Integration of PCA, HCA, and KNN to Evaluate Packaging and Storage Conditions for Red Bell Peppers

**DOI:** 10.1111/1750-3841.70367

**Published:** 2025-07-05

**Authors:** Eugénio da Piedade Edmundo Sitoe, Clara Mariana Gonçalves Lima, Jolanta Wawrzyniak, Matheus da Silva Mourão

**Affiliations:** ^1^ Department of Agricultural Engineering Universidade Estadual de Campinas Campinas São Paulo Brazil; ^2^ Department of Food Science Federal University of Lavras Lavras Minas Gerais Brazil; ^3^ Faculty of Food Science and Nutrition Poznań University of Life Sciences Poznań Poland; ^4^ Department of Food Science and Nutrition Campinas State University Campinas São Paulo Brazil

**Keywords:** *Capsicum annuum*, multivariate analysis, packaging, postharvest preservation, shelf life, storage

## Abstract

**ABSTRACT:**

Peppers (*Capsicum annuum* L.) are a vegetable that is widely cultivated in various regions of the world. Despite the economic importance of peppers, their commercialization is hindered by their limited postharvest durability, primarily due to moisture loss during storage. This study evaluated the effectiveness of different packaging methods and storage conditions in preserving the physicochemical and morphological quality of peppers during 21 days. Six treatments were tested, combining two types of packaging (thermo‐sealable and macro‐perforated) with two storage conditions (8°C/95% RH and 25°C/60% RH), plus an unpackaged control. Variables assessed included color, soluble solids, pH, pigments, dimensions, and mass loss. Data were analyzed using principal component analysis (PCA), hierarchical cluster analysis (HCA), and Kohonen neural networks (KNN). The first three principal components (PCs) explained 67.2% of total variance (PC1—40.88%, PC2—15.11%, PC3—11.17%). PC1 was strongly associated with mass and size losses (up to 73%), whereas PC2 and PC3 explained 77.4% of h* and 84.9% of C*, respectively. HCA and KNN revealed similar groupings. Samples stored at 8°C clustered together regardless of packaging, indicating minimal quality loss. At 25°C, unpackaged and macro‐perforated samples showed similar degradation. Thermo‐sealable packaging at 25°C formed a distinct cluster, indicating improved protection. This treatment also showed reduced quality losses, though not as effective as refrigeration. The agreement among PCA, HCA, and KNN confirms the reliability of findings. These results highlight the value of combining conservation strategies with multivariate tools to guide efficient, sustainable postharvest practices and extend shelf life in the pepper supply chain.

**Practical Application:**

This study proposes a solution for the horticultural industry by combining heat‐shrink packaging and refrigeration for pepper preservation. This method significantly reduces physical and biochemical losses, extends shelf life, and maintains quality. It has the potential to transform the logistics of production and distribution, delivering fresh, high‐quality peppers. The use of advanced techniques like PCA and neural networks enables more informed and efficient decision‐making, allowing for customized preservation strategies. This approach meets the growing demand for fresh food, offering a sustainable, cost‐effective alternative for postharvest preservation, and may provide a competitive advantage in the global market.

## 1 Introduction

The postharvest preservation of vegetables represents a significant challenge for contemporary agriculture, particularly in light of the persistent growth in global demand for fresh, safe, and high‐quality food (Elik et al. 2019; Adhikari and GC 2021). Among these vegetables, bell peppers (*Capsicum annuum* L.) are notable for their high nutritional value and wide culinary acceptance across various cultures (Sitoe et al. 2025). However, their high perishability imposes significant limitations on the production chain, with substantial losses during storage (Derry et al. 2017; Aşkin‐Uzel 2018; Dong et al. 2021; Zhang et al. 2021).

The shelf life of ripe peppers is, on average, only 10 d after harvest, a limited period that compromises their commercialization, especially for more distant markets (Popovsky‐Sarid et al. 2017). This limited shelf life is associated with accelerated water loss, which leads to early wilting, accelerates senescence, and affects critical sensory attributes such as firmness and texture (Maalekuu et al. 2005; Maalekuu et al. 2006). The interruption of the mother plant's water supply leads to a reduction in the fruit's water potential, which negatively impacts cell turgor and favors structural degradation (Galindo et al. 2004).

Postharvest losses have a direct impact on the sustainability of the agri‐food chain, leading to increased food waste and significant economic losses (Popovsky‐Sarid et al. 2017). Consequently, the implementation of effective preservation strategies is imperative to prolong shelf life, mitigate losses during distribution logistics, and ensure the delivery of products that meet the quality expectations of end consumers. The judicious selection of packaging systems and optimal storage conditions constitutes a pivotal step in this process (Adhikari and GC 2021).

Recent advances in food packaging technologies have emphasized the development of biodegradable films, active packaging, and intelligent systems to extend the shelf life of fresh produce (Atiwesh et al. 2021; Periyasamy et al. 2025; Yan et al. 2025). These innovations include the use of biopolymers, nanocomposites, and antimicrobial agents to reduce microbial spoilage and physiological degradation (Migliori et al. 2017; Khaneghah et al. 2018; Basumatary et al. 2022; Oliveira Filho et al. 2022; Tayel et al. 2024). While promising, such approaches often face economic and logistical constraints for commercial‐scale implementation, particularly for high‐volume vegetables such as peppers. In addition, biodegradable films, particularly those made from biopolymers such as chitosan and alginate, have been shown to be effective in preserving the quality of fruit and vegetables. Recent studies have shown that chitosan films, when incorporated with essential oils, have antimicrobial and antioxidant properties that extend the shelf life of produce (Popescu et al. 2022; Gunny et al. 2023; Soppelsa et al. 2023).

The antimicrobial properties of films composed of chitosan and glycerol were evaluated by Salvia‐Trujillo et al. (2015) using the disk diffusion methodology against strains of *Escherichia coli*, *Staphylococcus aureus*, and *Bacillus cereus*. In practical applications, coating strawberries with a solution containing 30% chitosan/glycerol has shown promising results in extending the shelf life of the fruit, promoting effective protection against bacterial and fungal microorganisms for a period of up to 7 days (Pavinatto et al. 2020). In addition to its antimicrobial properties, the treatment was found to preserve the desirable sensory characteristics of the product, including its appearance, texture, and flavor. Alginates activated with natural extracts have also been shown to maintain quality and extend the shelf life of horticultural products (Montone et al. 2022; Aly and Maraei 2024). These advances underscore the potential of biopolymer‐based films as sustainable alternatives to conventional packaging.

However, despite the promising results of biopolymeric films, they often face challenges such as higher production costs and reduced mechanical properties when compared to conventional materials. This highlights the continued relevance of low‐cost materials such as low‐density polyethylene (LDPE), as an alternative for the preservation of large quantities of vegetables. LDPE films offer mechanical strength, gas permeability, and the ability to effectively modulate the storage atmosphere, making them a practical solution for fresh produce such as peppers (Sitoe et al. 2025). Although biopolymers offer environmental benefits, LDPE remains a widely used and economically viable packaging material, particularly for preserving the freshness of high‐volume vegetables.

Thus, despite the momentum surrounding novel packaging technologies, optimizing conventional LDPE‐based systems continues to be a practical and relevant strategy in fresh vegetable preservation. This study aims to evaluate the applicability of both heat‐sealable packaging (LDPE) and conventional macro‐perforated packaging (MPP) to assess their effectiveness in extending the shelf life of red peppers. The study aimed to compare these materials in terms of their ability to extend product freshness, considering both sustainability and economic factors.

In addition to conventional physical and chemical preservation methods, the employment of statistical and computational tools for the analysis and prediction of fruit quality has emerged as a prominent approach. State‐of‐the‐art analytical techniques, including machine learning algorithms and multivariate statistical methods, have emerged as promising avenues for the interpretation of substantial volumes of experimental data and the optimization of postharvest strategies. Principal component analysis (PCA) and hierarchical cluster analysis (HCA) are two widely used techniques. These unsupervised methods have been firmly established in the scientific literature for analyzing physicochemical and sensory attributes (Barros et al. 2021; Albu et al. 2021).

These approaches facilitate the reduction of data dimensionality, the identification of dominant variables, and the revelation of hidden patterns in complex datasets (Bystrzanowska and Tobiszewski 2020; Chung et al. 2015). However, both PCA and HCA have limitations when applied to highly complex and nonlinear data, which can compromise the accuracy of the results in certain contexts (Bystrzanowska and Tobiszewski 2020; Chung et al. 2015). In this context, self‐organizing maps (SOM), also referred to as Kohonen neural networks (KNN), have been utilized as a complementary alternative on an increasingly broader scale. These networks possess the capacity to project multidimensional data into lower dimensional spaces while preserving topological relationships. Furthermore, they demonstrate resilience to noise and inconsistencies in the data (Kohonen 1982; Wehrens and Kruisselbrink 2018). The applications of SOM have expanded in various scientific domains, including food quality assessment (Shi et al. 2022; Koudenoukpo et al. 2021; Caetano et al. 2022; Drgan and Bajželj 2021). However, there is a paucity of studies that systematically integrate PCA, HCA, and KNN to investigate the postharvest quality of peppers stored under different conditions.

In addressing this gap, the present study sought to assess the applicability of PCA, HCA, and KNNs in the classification and ranking of storage methods based on their effectiveness in preserving the freshness of red peppers, with a focus on physicochemical parameters. The integration of these multivariate techniques aims to identify the most promising patterns and strategies for preservation, contributing both to the improvement of postharvest management and to the advancement of technological innovation in the horticultural sector.

## 2 Materials and Methods

### 2.1 Raw Material

The study used red bell peppers (*C. annuum* L.) harvested at the commercial ripeness stage, characterized by approximately three‐quarters red coloration. The peppers were purchased from a farm located in Campinas, São Paulo, Brazil. After harvesting, the fruit were packed in ventilated plastic boxes and transported to the laboratory at a controlled temperature of 20°C to minimize physiological and mechanical deterioration during transport.

In the laboratory, the fruits were initially characterized by carefully selecting units with a uniform degree of ripeness and shape, free from mechanical damage, rot, or signs of visible disease. To ensure experimental consistency, only peppers with similar size (10% in length and width), color uniformity, and firmness were included. The fruits were visually inspected and manually sorted by trained personnel under standardized lighting conditions. The selected peppers were then subjected to the following analyses: evaluation of surface color, determination of total soluble solids content (°Brix), measurement of hydrogen potential (pH), quantification of pigments (chlorophyll *a*, chlorophyll *b*, anthocyanins, and carotenoids), measurement of orthogonal dimensions (length, width, and thickness), and determination of the initial mass of each fruit using a precision analytical balance.

### 2.2 Addition of Packaging to the Red Bell Peppers

#### 2.2.1 Application of Thermo‐Sealable Packaging (TSP)

The thermo‐sealable packaging (TSP) (LDPE) was applied using a permeable film capable of conforming to the product when exposed to heat. Initially, the peppers were wrapped in thermo‐sealing film, followed by a heating process at 150°C for 10 s, which ensured that the film adhered completely to the surface of the fruit. The procedure was carried out using MINIPACK‐TORRE S.p.A. equipment (Dalmine, BG, Italy). After packaging, the peppers were visually inspected to ensure that the film adhered uniformly to the surface, with no air bubbles or wrinkles that could compromise the integrity of the packaging and the protection of the fruit during storage.

#### 2.2.2 Application of Macro‐Perforated Packaging (MPP)

The MPP was applied using an FM 300 horizontal flowpack machine (São Paulo, Brazil). Initially, the machine underwent a calibration process to ensure precision in cutting and sealing the film. This process involved the configuration of specific parameters, including temperature, pressure, and sealing time, which were determined on the basis of the properties of the film material and the dimensions of the peppers. Each bell pepper was meticulously inserted into the machine's feeder, where it was automatically enveloped in the film, resulting in a cylindrical enclosure that ensured complete sealing of the peppers. The longitudinal sealing was carried out using heated bars, which melted the ends of the film, resulting in a continuous, airtight package. Subsequently, the machine proceeded to carry out the transverse sealing and cutting of the film, thereby effecting the separation of each package into its individual units. The perforation of the packages, essential for facilitating gas exchange during storage, was accomplished by means of a mechanical punching system. This system was meticulously calibrated to generate perforations of a uniform size, distributed across the surface of each package. After the packaging process, a rigorous inspection was conducted to ascertain the integrity of the seal and the conformity of the macro‐holes. This inspection utilized preestablished quality criteria, such as tightness tests and visual assessment.

### 2.3 Experimental Planning and Storage Conditions for Peppers

**FIGURE 1:**
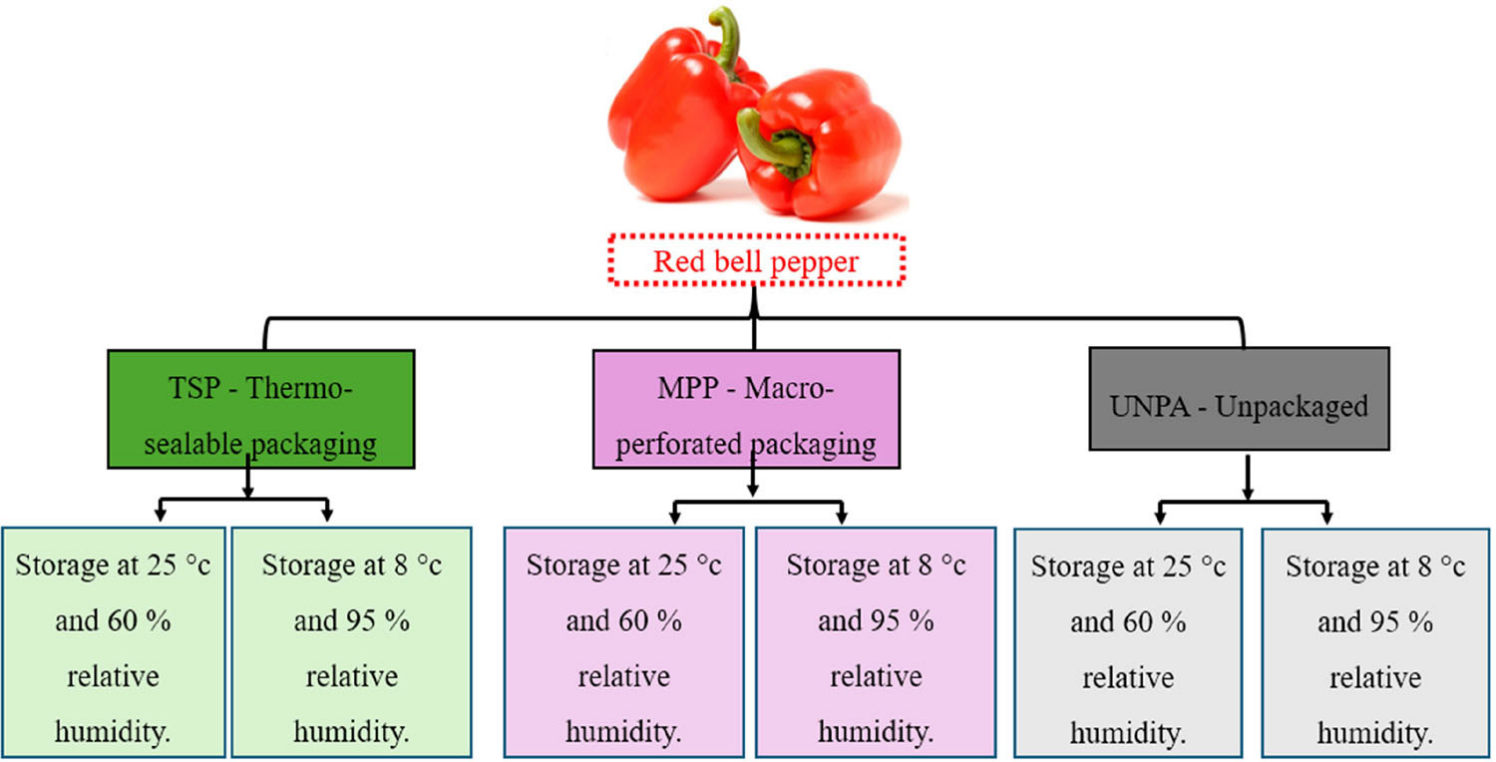
Treatments evaluated in the experimental trial.

Figure 1 illustrates the experimental scheme adopted in this study, which followed a completely randomized design (CRD), consisting of six different treatments, each with three replications. In each repetition, 10 peppers were evaluated, totaling 30 units per treatment. Randomization was performed using a computerized random number generator to assign fruit to treatment groups, ensuring that each fruit had an equal chance of being allocated to any condition, thereby reducing selection bias.

The experiment included two different storage conditions applied to peppers packed with TSP or MPP, as well as a control group made up of unpacked peppers (UNPA). The storage conditions included combinations of temperatures of 25°C and 8°C with relative humidities of 60% and 95%, as specified for each treatment in Figure 1. Relative humidity was controlled using saturated salt solutions inside the storage chambers, following standard protocols (sodium chloride [NaCl] for 75% and potassium nitrate [KNO_3_] for 95%). Environmental conditions were continuously monitored using digital hygrometers (model HMT337, Vaisala Inc., Helsinki, Finland), with a measuring range of 0%–100% RH and accuracy of ±2%, as specified by the manufacturer. Instruments were calibrated prior to use according to the manufacturer's guidelines.

### 2.4 Quality of Peppers During Storage

The quality of the peppers was assessed at regular intervals of 7 d over a total storage period of 21 d (0, 7, 14, and 21 d). At each interval, the following physicochemical and morphological analyses were conducted: bell pepper color, total soluble solids content (%), pH, pigment content (chlorophyll *a*, chlorophyll *b*, anthocyanins, and carotenoids), fruit dimensions (length, width, and thickness), and mass loss. All determinations were performed in triplicate.

#### 2.4.1 Instrumental Analysis of Bell Pepper Color

The color of the peppers was assessed by direct measurements of the reflectance of the L*, a*, and b* coordinates using a CR410 colorimeter (Konica Minolta, Osaka, Japan). On the basis of the reflectance values obtained from the surface of the peppers, the following parameters were calculated: luminosity (L*), color tone or hue angle (h*, Equation 1), color saturation or chroma (C*, Equation 2), and total color difference (ΔE*, Equation 3) (Giusti et al. 2024; Dutta and Nath 2024):
(1) $$\;C^{*}=\sqrt{a^{*2}+b^{*2}}$$
(2) $$h^{*}=\mathrm{arctang}\left(\frac{b^{*}}{a^{*}}\right)$$
(3) $$\Delta E^{*}=\sqrt{\left(L^{2}-L_{0}^{*}\right)+{\left(a^{*}-a_{0}^{*}\right)}^{2}+{\left(b^{*}-b_{0}^{*}\right)}^{2}}$$

where $L_{0}^{*},a_{0,}^{*}$ and $b_{0}^{*}$ are the coordinates obtained from the control bell pepper (unpackaged).

#### 2.4.2 Total Soluble Solids

Total soluble solids were determined using a digital refractometer model MA871 (Rocky Mount, North Carolina, USA), according to the protocols of the Adolfo Lutz Institute (IAL 2008). To obtain the extract, previously fragmented and crushed bell pepper samples were filtered through sterile cotton gauze (porosity of approximately 100–200 µm), and an aliquot of the extracted liquid was immediately transferred to the optical sensor of the equipment. The readings were performed in triplicate, with the results expressed in °Brix (%).

#### 2.4.3 Hydrogen Potential (pH)

The pH of the peppers was measured using a bench pH meter (model PH2500, Laborchemiker, Curitiba‐PR, Brazil), which had been previously calibrated with standard buffer solutions of pH 4.0, 7.0, and 10.0 before each series of readings was taken. The samples were prepared by first cutting the fruit into small pieces using a stainless steel knife that had been previously sterilized. The samples were then ground in a manual stainless steel grinder (model Maxx BMX355P, Britânia Eletrônicos SA, Joinville‐SC, Brazil) until a homogeneous pulp was obtained. The pulp was then subjected to vigorous shaking and filtration using sterile cotton gauze with an approximate pore size of 100–200 µm to yield the liquid extract. The pH was measured by directly immersing the electrode in the filtered solution and waiting for the reading to stabilize. Each sample was analyzed in triplicate, and the results were expressed as the average of the three values obtained.

#### 2.4.4 Pigments

The pigment contents (chlorophyll *a*, chlorophyll *b*, anthocyanins, and carotenoids) were quantified using spectrophotometry, following the method described by Sims and Gamon (2002). To extract the pigments, 1 g of the liquid sample was mixed with 3 mL of acetone buffered with TRIS–HCl (80:20 v, pH 7.8, 0.2 M). The resulting mixture was homogenized using a Vortex mixer (Model AP‐56, PHOENIX, Vila Mariana, SP, Brazil), followed by centrifugation at 820 × *g* for 5 min. The supernatants obtained were subjected to absorbance measurement in a UV–VIS spectrophotometer (Q798U2M, QUIMIS, Brazil) at wavelengths of 470, 537, 647, and 663 nm. The absorbance values were then converted into the concentrations of chlorophyll *a* (Equation 4), chlorophyll *b* (Equation 5), anthocyanins (Equation 6), and carotenoids (Equation 7), expressed in mg per 100 g of sample (Equation 8):

(4) $$\mathrm{Cla}=\left(0.01373A_{663}\right)-\left[\left(0.000897A_{537}\right)-\left(0.003046A_{647}\right)\right]$$

(5) $$\mathrm{Clb}=\left(0.02405A_{647}\right)-\left[\left(0.004305A_{537}\right)-\left(0.005507A_{663}\right)\right]$$

(6) $$\mathrm{Ant}=\left(0.08173A_{537}\right)-\left[\left(0.00697A_{647}\right)-\left(0.002228A_{663}\right)\right]$$

(7) $$\mathrm{Car}=\left\{A_{470}-\left[17.1\left(\mathrm{Cla}+\mathrm{Clab}\right)\right]-\left(9.479\;\mathrm{Ant}\right)\right\}\times \frac{1}{119.26}$$

(8) $$\mathrm{Pigment}\;\mathrm{Content}=\left[M\right]\times \frac{v}{m}\times M\times 0.1$$

where 𝐶𝑙𝑎, 𝐶𝑙𝑏, 𝐴𝑛𝑡, and 𝐶𝑎𝑟 are molar concentration of chlorophyll *a* and *b*, anthocyanin, and carotenoid, respectively (µmol mL^−1^); $A_{\lambda }$ is the absorbance of the pigment extraction at wavelength *λ*; 𝑃𝑖𝑔𝑚𝑒𝑛𝑡 𝐶𝑜𝑛𝑡𝑒𝑛𝑡 is mg pigment 100 g^−1^ extract; [𝑀] is molar concentration (µmol m^−1^); 𝑚 is sample weight (g); 𝑉 is pigment extraction volume (mL); 𝑀 is molar mass (893. 5 g mol^−1^ for chlorophyll *a*, 907.5 g mol^−1^ for chlorophyll *b*, 449.2 g mol^−1^ for anthocyanin, and 550.0 g mol^−1^ for carotenoid).

#### 2.4.5 Dimensions of Peppers and Mass Loss

The dimensions of the peppers were evaluated in terms of their orthogonal axes: length, width, and thickness. For this analysis, one bell pepper from each repetition was randomly selected on the first day of evaluation and identified with an asterisk. Measurements were taken using a digital caliper with a precision of 0.05 mm, and the dimensions of the orthogonal axes were recorded throughout the storage period. The results were expressed in millimeters (mm).

Mass loss was determined by calculating the difference between the initial mass of each sample at the start of the experiment (0 day) and the masses recorded after 7, 14, and 21 d of storage, according to Equation (9). Weighing was carried out using a digital semi‐analytical balance, model BK 8000 (Gehaka, São Paulo, Brazil), accurate to 0.01 g. The results were expressed in percentage terms (%), reflecting the variation in mass over the different storage intervals:

(9) $$\mathrm{WLt}\;\left(\%\right)=\frac{W_{0}-W_{t}}{W_{0}}\times 100$$

where the percentage mass loss is represented by WLt (%). The value of *W*_₀_ corresponds to the initial mass at 0 day, and the value of *W_t_* refers to the mass of the sample on the evaluated day.

### 2.5 Statistical Analysis

The impact of different packaging methods and storage conditions on the quality of red bell peppers, assessed through physicochemical parameters, was analyzed using multivariate statistical techniques in Statistica 13.3 software (StatSoft, Tulsa, OK, USA). The analytical workflow comprised (1) PCA, (2) HCA, and (3) KNNs. Prior to these analyses, the relevance of each physicochemical parameter (independent variables) to packaging methods and storage conditions (categorical dependent variables) was assessed using Cramér's *V* coefficient. Variables with statistically significant associations (*α* = 0.005) were retained for further analysis.

PCA was initially applied to reduce data dimensionality and identify key variables contributing to sample differentiation. To evaluate the adequacy of the dataset for PCA, Bartlett's test of sphericity and the Kaiser–Meyer–Olkin (KMO) measure of sampling adequacy were performed. Although not strictly mandatory, the KMO test was considered a widely accepted diagnostic tool that assesses the proportion of variance that may be attributed to underlying common factors. A high KMO value indicated that PCA would likely yield reliable and distinct components, thus ensuring the robustness of the dimension reduction process. Therefore, its computation served as an important preliminary step in validating the applicability of PCA to the dataset. The number of principal components (PCs) retained was defined using Kaiser's eigenvalue criterion (>1) and Cattell's scree plot. The PCA output included factor scores for each treatment, used to assess the spatial distribution of samples in relation to quality preservation. Loadings were examined to interpret the contribution of each variable to the PCs.

Regarding the choice of PCA over other multivariate techniques such as partial least squares discriminant analysis (PLS‐DA) or support vector machines (SVM), PCA was chosen primarily for its suitability in the exploratory phase of this study. PLS‐DA and SVM are valuable for classification tasks, but they are generally more suitable when there is a predefined dependent variable for prediction or classification. As the primary aim of this study was to explore the underlying structure of the dataset and reduce its dimensionality, PCA was considered the most appropriate technique. PCA allows for a comprehensive analysis of the variance in the data without requiring a predefined response variable, which aligns with the objectives of understanding the general trends and relationships in the dataset. Although PLS‐DA and SVM could potentially increase classification efficiency, their application would necessitate additional steps, such as defining a categorical outcome variable, which was outside the scope of the present analysis. Therefore, PCA was sufficient and robust for identifying key factors influencing the quality of the red bell peppers under the different conditions of packaging and storage.

HCA was then performed using Ward's linkage method, which minimizes the total within‐cluster variance, to group treatments with similar effectiveness. A dendrogram was constructed to visualize treatment similarity. The classification power of treatments was evaluated using Kohonen SOM, a type of unsupervised neural network. Multiple network topologies were tested using rectangular grids with varying neuron counts in the output layer. The networks were trained using the Kohonen learning algorithm with Gaussian neighborhood functions over 10,000 epochs. Model accuracy was assessed using quantization error (QE), representing the average distance between input vectors and their corresponding best matching units (BMUs). Lower QE values indicated better mapping performance and classification accuracy.

## 3 Results and Discussion

### 3.1 Quality of Peppers During Storage

The evaluation of the physicochemical parameters of the peppers in storage revealed that the characteristics most influenced by the packaging methods and storage conditions were color, mass loss, and dimensional variations in the fruit (Tables 1, 2, 3). The data obtained demonstrated that fruit stored under similar temperature and relative humidity conditions exhibited statistically similar averages, irrespective of the type of packaging employed, thereby forming homogeneous groups. It was found that, in general, the treatments stored at 8°C and 90% RH showed superior physicochemical characteristics compared to the treatments stored at 25°C and 60% RH, except for the samples packed with heat shrink packaging (TSP 25 60), which performed similarly to the refrigerated treatments in various parameters (C*, TSS, pH, losses in length, width, thickness, and mass).

**TABLE 1:** Mean values of color parameters (L*, C*, h*, and ΔE*) of red peppers under different packaging methods (TSP, MPP, and UNPA) and storage conditions (8°C and 25°C; 60% and 95% RH).

**Treatment**	L*	C*	h*	ΔE*
UNPA 8 90	45.81^b^	34.32^d^	23.14^a^	6.98^a^
MPP 8 90	43.04^b^	31.81^bcd^	23.83^a^	7.28^a^
TSP 8 90	45.43^b^	33.26^cd^	23.30^a^	5.46^a^
UNPA 25 60	34.42^a^	28.25^a^	26.21^ab^	27.79^c^
MPP 25 60	32.98^a^	29.58^ab^	29.66^b^	19.38^b^
TSP 25 60	34.06^a^	30.75^abc^	30.80^b^	20.96^b^

*Note*: Different superscript letters denote significant differences (*p* < 0.05) among the pepper samples. Means sharing the same letter index do not differ significantly at *α* = 0.05 and are classified within the same homogeneous group. ΔE*—color difference; h*—hue angle; C*—color saturation; and L*—luminosity.

Abbreviations: MPP, macro‐perforated packaging; TSP, thermo‐sealable packaging; UNPA, unpackaged.

**TABLE 2:** Mean values of chemical parameters (SST, pH, chlorophyll *a* and *b*, anthocyanins, and carotenoids) of red peppers subjected to different packaging methods (TSP, MPP, and UNPA) and stored under varying temperature (8°C and 25°C) and relative humidity conditions (60% and 95%).

Treatment	SST (%)	pH	Chlorophyll *a*	Chlorophyll *b*	Anthocyanin	Carotenoids
UNPA 8 90	4.97^b^	4.86^a^	0.61^a^	1.26^a^	3.04^ab^	0.71^a^
MPP 8 90	5.76^a^	4.94^ab^	0.49^a^	1.12^a^	2.51^ab^	0.80^a^
TSP 8 90	5.73^a^	5.00^b^	0.86^a^	1.78^a^	3.20^b^	0.74^a^
UNPA 25 60	5.26^ab^	4.91^a^	0.29^a^	2.63^a^	1.31^a^	0.66^a^
MPP 25 60	5.03^ab^	4.88^a^	0.63^a^	1.71^a^	2.49^ab^	0.92^a^
TSP 25 60	5.42^ab^	4.94^ab^	0.24^a^	1.83^a^	1.37^a^	0.66^a^

*Note*: Different superscript letters denote significant differences (*p* < 0.05) among the pepper samples. Means sharing the same letter index do not differ significantly at *α* = 0.05 and are classified within the same homogeneous group.

Abbreviations: MPP, macro‐perforated packaging; pH, hydrogenonic potential; SST, soluble solids content; TSP, thermo‐sealable packaging; UNPA, unpackaged.

**TABLE 3:** Mean values of losses in length, width, thickness, and mass of red peppers subjected to different packaging methods and stored under varying temperature (8°C and 25°C) and relative humidity conditions (60% and 95%).

Treatment	Length loss (%)	Width loss (%)	Thickness loss (%)	Mass loss (%)
UNPA 8 90	7.92^ac^	12.38^b^	8.58^ab^	3.03^a^
MPP 8 90	11.92^a^	8.91^ab^	10.16^ab^	3.71^a^
TSP 8 90	0.61^b^	9.83^ab^	12.25^b^	2.00^b^
UNPA 25 60	35.62^d^	24.16^c^	19.57^c^	8.68^d^
MPP 25 60	14.29^a^	22.11^c^	42.66^d^	7.31^c^
TSP 25 60	4.62^bc^	7.16^a^	4.79^a^	3.11^a^

*Note*: Different superscript letters denote significant differences (*p* < 0.05) among the pepper samples. Means sharing the same letter index do not differ significantly at *α* = 0.05 and are classified within the same homogeneous group.

Abbreviations: MPP, macro‐perforated packaging; TSP, thermo‐sealable packaging; UNPA, unpackaged.

This emphasizes the significance of heat shrink packaging (TSP) as an effective barrier against moisture loss and gas exchange, even in unfavorable environmental conditions. The packaging limits the transpiration and respiration of the fruit, thereby helping to maintain its quality after harvest. The impact of temperature on the stability of fruit preservation was also examined, with Sitoe et al. (2025) demonstrating that unpackaged peppers stored at 25°C and 60% RH exhibited significantly higher mass losses compared to those packaged with heat‐sealable packaging. Notably, heat‐sealable packaging was found to be effective in reducing mass loss at both 20°C and 8°C, as reported in this study and by other authors, thereby underscoring the significance of this technology in extending the shelf life of sensitive vegetables.

The most significant variation between the means of the groups analyzed was observed precisely in the parameters related to mass loss and the dimensions of the peppers (length, width, and thickness), demonstrating that these attributes are highly sensitive to both the type of packaging and the storage temperature (Table 3). The unpackaged peppers (UNPA) exhibited the most significant mass loss, irrespective of the storage temperature, indicating an absence of a barrier to transpiration and water loss to the environment. These outcomes align with those reported by Alam et al. (2021) and Umeohia and Olapade (2024), who underscored the pivotal role of packaging in regulating respiration rate and mitigating water stress during the postharvest phase. Conversely, the lowest mass loss was observed in fruit packed in TSP, followed by those in MPP, which exhibited an intermediate level of efficiency.

**FIGURE 2:**
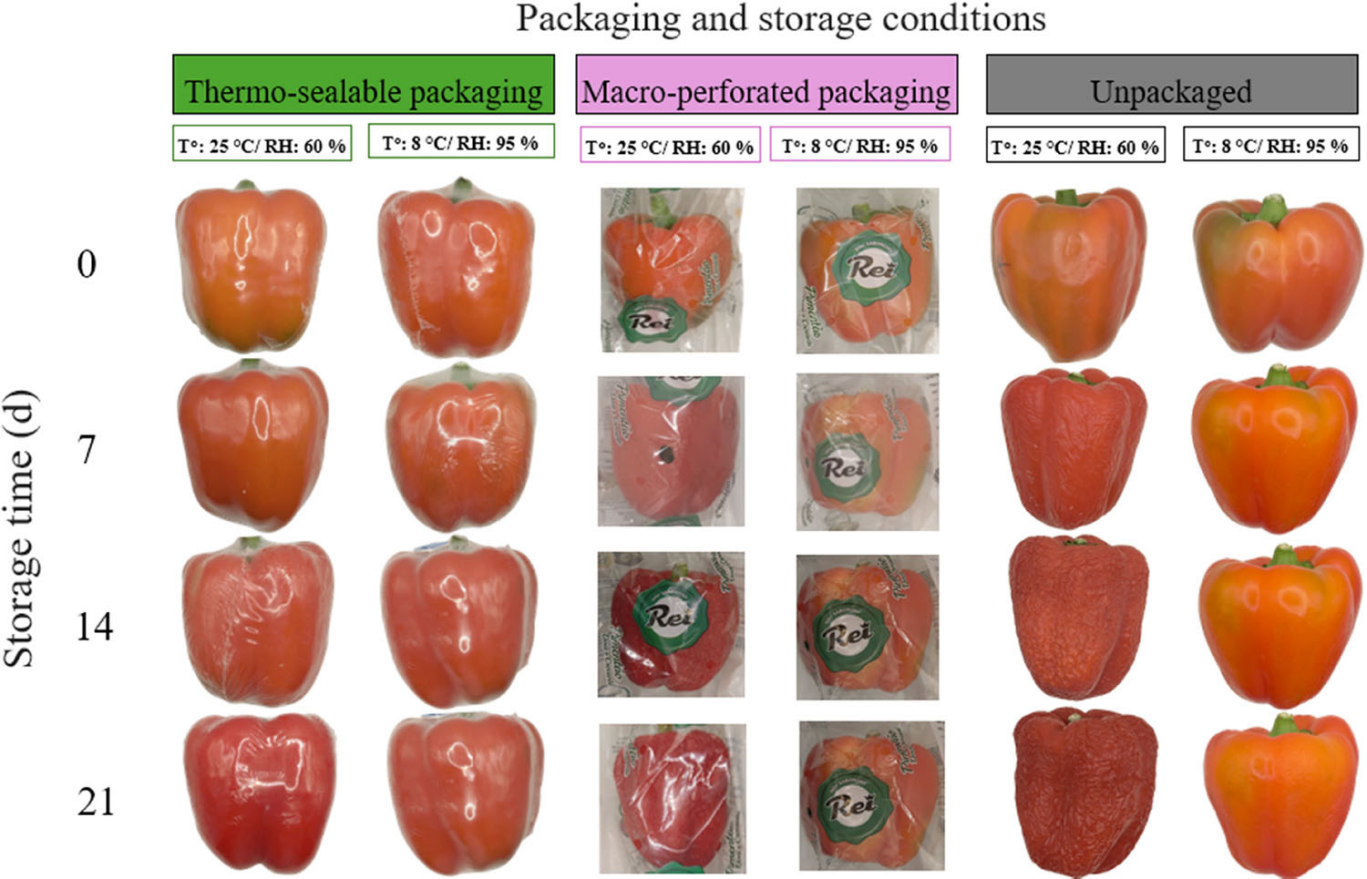
Visual appearance of peppers subjected to different types of packaging (thermo‐sealable, macro‐perforated and unpackaged) and stored for 21 days in two temperature (*T*°) and relative humidity (RH) conditions: 25°C/60% and 8°C/95%.

Furthermore, the temperature at which the peppers were stored had a significant impact on their visual quality (Figure 2). For instance, peppers stored at 25°C exhibited a more substantial decline in mass and physical dimensions, whereas those maintained at 8°C demonstrated a superior preservation of their original characteristics. The reduction in respiration rate and metabolic activity at lower storage temperatures (Fawole and Opara 2013) is associated with a decrease in water and organic reserves lost from the food matrix. However, the data demonstrated that even under ambient temperature conditions (20°C), heat shrink packaging (TSP 25 60) was able to mitigate these losses, thus emphasizing its potential as a viable alternative in contexts where refrigerated storage is not feasible.

The formation of a distinct group by the TSP 8 90 samples in the case of mass loss and length indicates that this combination of environmental factors and type of packaging was the most effective in preserving the physical integrity of the fruit. In addition to the role of packaging, temperature and relative humidity have been identified as critical factors in maintaining cell structure, thereby delaying the process of wilting and tissue collapse, which is characteristic of postharvest senescence (Sitoe et al. 2025). The grouping of TSP 25 60 with the refrigerated treatments also reveals the compensatory potential of TSP in the face of thermal stress, which can be exploited in storage systems where refrigeration is not economically viable.

Finally, the lower variability observed in the pigments (chlorophyll *a*, chlorophyll *b*, and carotenoids) between the different groups tested suggests a greater stability of these bioactive compounds in the first 3 weeks of storage, regardless of the packaging or thermal condition (Table 2). This relative stability may be associated with the lipophilic nature of carotenoids and the intracellular location of chlorophylls, which remain protected within chloroplasts during the initial stages of storage, especially when the tissues do not show signs of advanced senescence (Mazza and Miniati 1993; Solovchenko et al. 2019). Beyond structural protection, the degradation of chlorophylls is tightly regulated by enzymatic pathways, primarily involving chlorophyllase, which catalyzes the hydrolysis of chlorophyll to chlorophyllide and phytol. The activity of chlorophyllase is temperature‐dependent and tends to increase under heat stress and oxidative conditions, promoting faster pigment loss (Gao et al. 2019; Jiang et al. 2020). In later stages of degradation, chlorophyllide is converted into pheophorbide by Mg‐dechelatase, followed by cleavage by pheophorbide a oxygenase (PAO), forming noncolored catabolites (González‐Aguilar 2017). These sequential enzymatic reactions constitute the PAO/phyllobilin pathway, which plays a major role in senescence and postharvest degradation. Moreover, chlorophyll *a* is generally more prone to degradation than chlorophyll *b* due to differences in its structure and binding to photosynthetic proteins (Matsuda et al. 2016; Pareek et al. 2017; Tanaka and Ito 2025). Thus, the observed pigment stability may reflect not only favorable environmental conditions but also limited activation of these catabolic pathways. A study by Park et al. (2018) indicates that temperatures above 12°C, when associated with low humidity, accelerate the degradation of chlorophylls and the enzymatic conversion of carotenoids, which tends to occur after prolonged storage periods. Consequently, although the pigments exhibited stability during the evaluation period, the utilization of effective packaging, such as heat shrink, and refrigerated storage can further delay the onset of their degradation, thereby preserving the nutritional value and visual appeal of the peppers.

In the case of anthocyanins, the synthesis of which has been shown to be induced by oxidative or thermal stress (Zaidi et al. 2019; Oancea 2021), the slight variation observed between the treated groups indicates that there was no significant accumulation or degradation during storage. This finding serves to reinforce the hypothesis that the conditions tested are effective in protecting the fruit plant tissue. Furthermore, the use of packaging that limits exposure to oxygen, such as heat shrink, has been demonstrated to help limit the oxidation of these sensitive molecules (Sitoe et al. 2025). Consequently, the combination of low temperature, high relative humidity, and the utilization of packaging with a robust gas barrier not only mitigates physical losses but also plays a pivotal role in the biochemical preservation of photosynthetic pigments and antioxidants. This is of paramount importance for ensuring the maintenance of the sensory and functional quality of the fruit.

### 3.2 Statistical Interpretation of the Effectiveness of Protective Treatments of Red Pepper Using Multivariate Analysis and Machine Learning

To develop tools for the automatic classification of the examined pepper samples and to rank the storage methods and conditions based on their practical effectiveness, three different data analysis techniques were applied. The analyses were performed using nine selected pepper quality parameters (color factors, pH, and the loss of mass and dimensions), whose strength of association with the applied packaging methods and storage conditions was confirmed by the computed values of Cramér's *V* coefficient. The results of Cramér's *V* analysis, presented in Table 4, highlighted that the percentage of mass losses and changes in pepper dimensions were the primary factors most strongly related to the applied packaging method and storage conditions; color parameters and pH level followed (*p* < 0.005).

**TABLE 4:** Cramér's *V* coefficient values and significance levels indicating the relationship between the analyzed variables and the applied packaging methods and storage conditions of red pepper.

Variable	Cramer's *V*	*p* value	
Width loss (%)	0.471	<0.00001	^*^
Length loss (%)	0.510	<0.00001	^*^
Thickness loss (%)	0.528	<0.00001	^*^
Mass loss (%)	0.612	<0.00001	^*^
ΔE*	0.528	<0.00001	^*^
L*	0.434	<0.00001	^*^
C*	0.338	<0.00001	^*^
h*	0.273	0.00444	^*^
pH	0.259	0.00497	^*^
Anthocyanins (mg 100 g^−1^)	0.213	0.02393	
SST (%)	0.235	0.05065	
Chlorophyll *b* (mg 100 g^−1^)	0.212	0.09860	
Carotenoids (mg 100 g^−1^)	0.220	0.15507	
Chlorophyll *a* (mg 100 g^−1^)	0.168	0.43449	

*Note: p* value: probability value of the statistical test at a significance level of *α* = 0.005. ΔE*—color difference; h*—hue angle; C*—color saturation; L*—luminosity.

Abbreviations: pH, hydrogenonic potential; SST, soluble solids content.

* Significant at the 5% significance level (p < 0.05).

**FIGURE 3:**
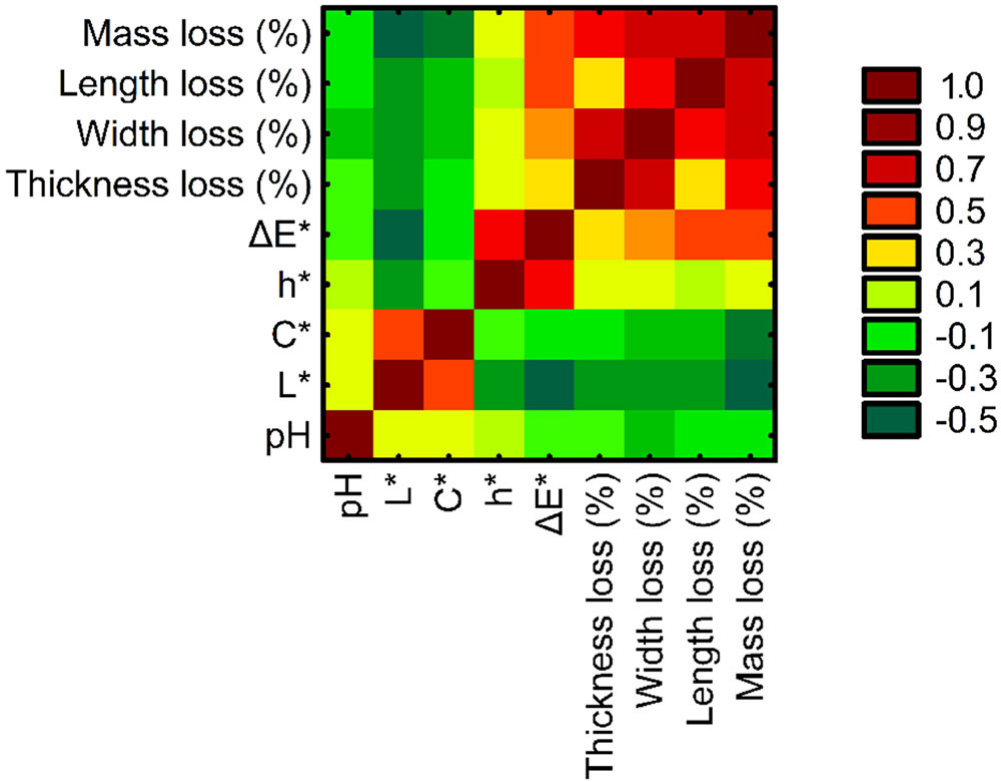
Heatmap of Pearson's correlation coefficients between the physicochemical parameters of the examined pepper. ΔE*—color difference; h*—hue angle; C*—color saturation; L*—luminosity. pH, hydrogenonic potential.

Since the first of the applied clustering analyses, PCA is intended for the analysis of phenomena or objects characterized by interdependent variables, and before its implementation, the multivariate correlation matrix of selected pepper quality factors was also examined (Figure 3). This preliminary analysis provided insight into potential multicollinearity among the variables and helped assess the strength and direction of dependencies among them. Additionally, it confirmed the suitability of PCA for dimensionality reduction by identifying the most significant variables contributing to variance in the dataset.

The significant relationships and thus redundancy among the variables were further confirmed by Bartlett's test (*p* < 0.0001). This result suggested that the considered variables could be effectively represented by a smaller set of PCs (Bartlett 1951; Shrestha 2021). Following Bartlett's test, the KMO criterion, which compares partial correlation coefficients between variables in a two‐dimensional space, was also used to assess the adequacy of PCA (Cerny and Kaiser 1977; Kaiser 1970). The KMO value ranges from 0 to 1, where values between 0.5 and 1.0 justify the use of PCA, whereas values below 0.5 suggest that variable reduction may be limited (Shrestha 2021). In our study, the KMO criterion value of 0.75 further supported the suitability of factor analysis.

**FIGURE 4:**
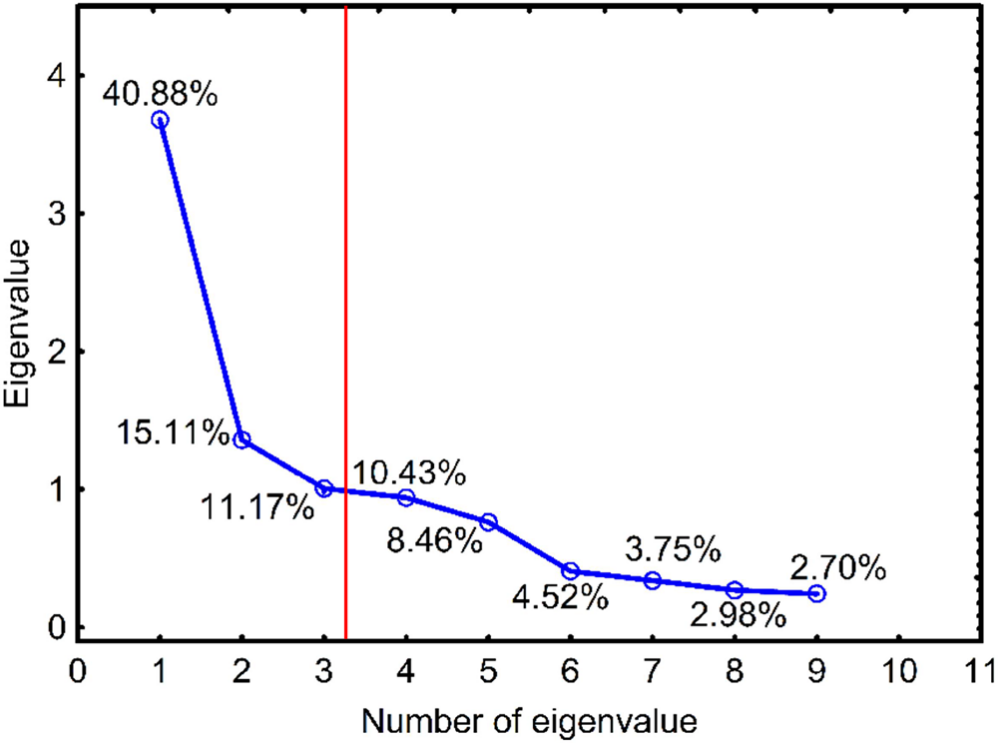
A Cattell scree plot displaying the estimated number of principal components in a PCA analysis.

After verifying the assumptions, PCA was performed to identify the subsequent PCs. The optimal number of PCs was established using the Kaiser and Cattell criteria. The Kaiser criterion indicated that the first three PCs should be considered, based on the rule that only components with eigenvalues (reflecting the amount of total variance explained by each component) greater than 1 could be deemed significant (Shrestha 2021). The Cattell criterion, a graphical method for determining the number of PCs based on the “elbow” rule, also suggested that the first three components, which preceded a sharp decline in eigenvalues in the scree plot (Figure 4), were sufficient to represent the total variance in the data (Cattell 1978; Shrestha 2021).

The three determined PCs explain more than 67.2% of the data variability (PC1—40.88%, PC2—15.11%, PC3—11.17%). The acceptable level of explained variance depends on the specific problem context; however, it is generally recommended that the extracted factors explain at least 60% of the variance. In this context, the sum of data variability explained by the first three PCs suggests that they should be able to simplify the dataset while retaining key information (by capturing the main patterns and structures in the data).

**FIGURE 5:**
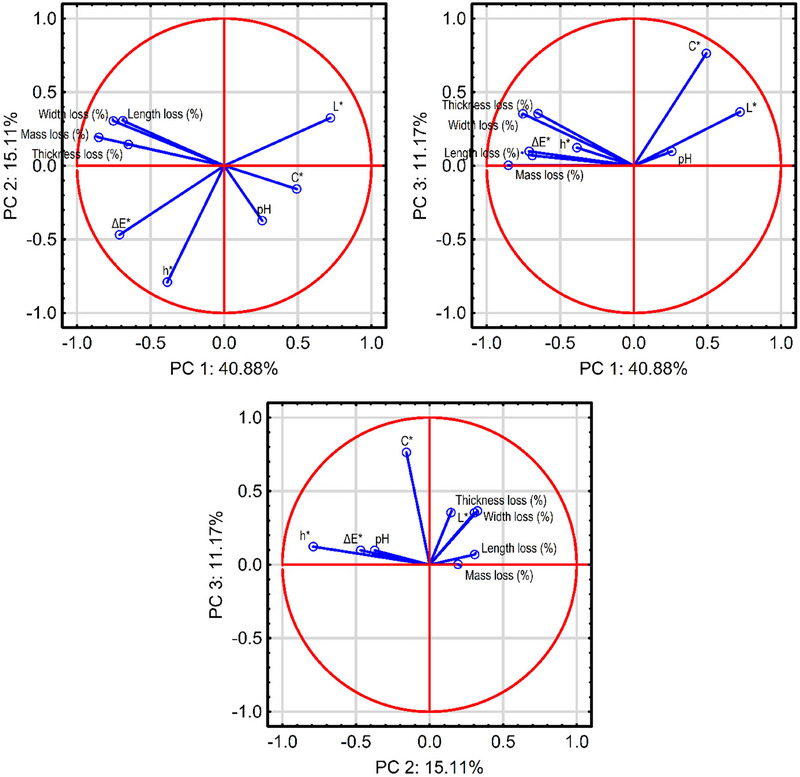
Factor maps of the variables, showing the strength and direction of each red pepper quality parameter contribution to a given principal component determined in the PCA analysis. PC, Principal component.

Analyzing the loadings of individual variables on the first component (PC1), it can be concluded that this component primarily reflects losses in mass and dimensions (length, width, thickness) of the pepper, explaining 73%, 47%, 57%, and 42% of their total variability, respectively, as well as in L* (luminosity) and ΔE* (total color difference), explaining 52% and 51% of their variance (Figure 5). The second component was correlated with h* (color tone or hue angle), whereas the third component was associated with C* (color saturation or chroma), explaining 77.4% and 84.9% of the variability of the mentioned variables, respectively.

Although the first three PCs account for 67.2% of the total variance, it is important to acknowledge that the remaining 32.8% may contain secondary biological variation or noise that is not directly related to the primary physicochemical changes occurring during storage. The purpose of the PCA in this context was to reduce dimensionality and reveal the dominant patterns associated with postharvest quality loss in peppers, especially related to mass, size, and color parameters. The key variables of interest were strongly represented in the retained components, as demonstrated by their high loadings, indicating that essential biological information was effectively captured. Although including additional components could mathematically increase the explained variance, this would likely introduce more noise and reduce interpretability, without providing substantial new insight into the principal quality‐related processes. Therefore, the retention of three components was considered adequate for the biological interpretation of the dataset, allowing for a reliable summary of the most meaningful variance associated with storage conditions.

**FIGURE 6:**
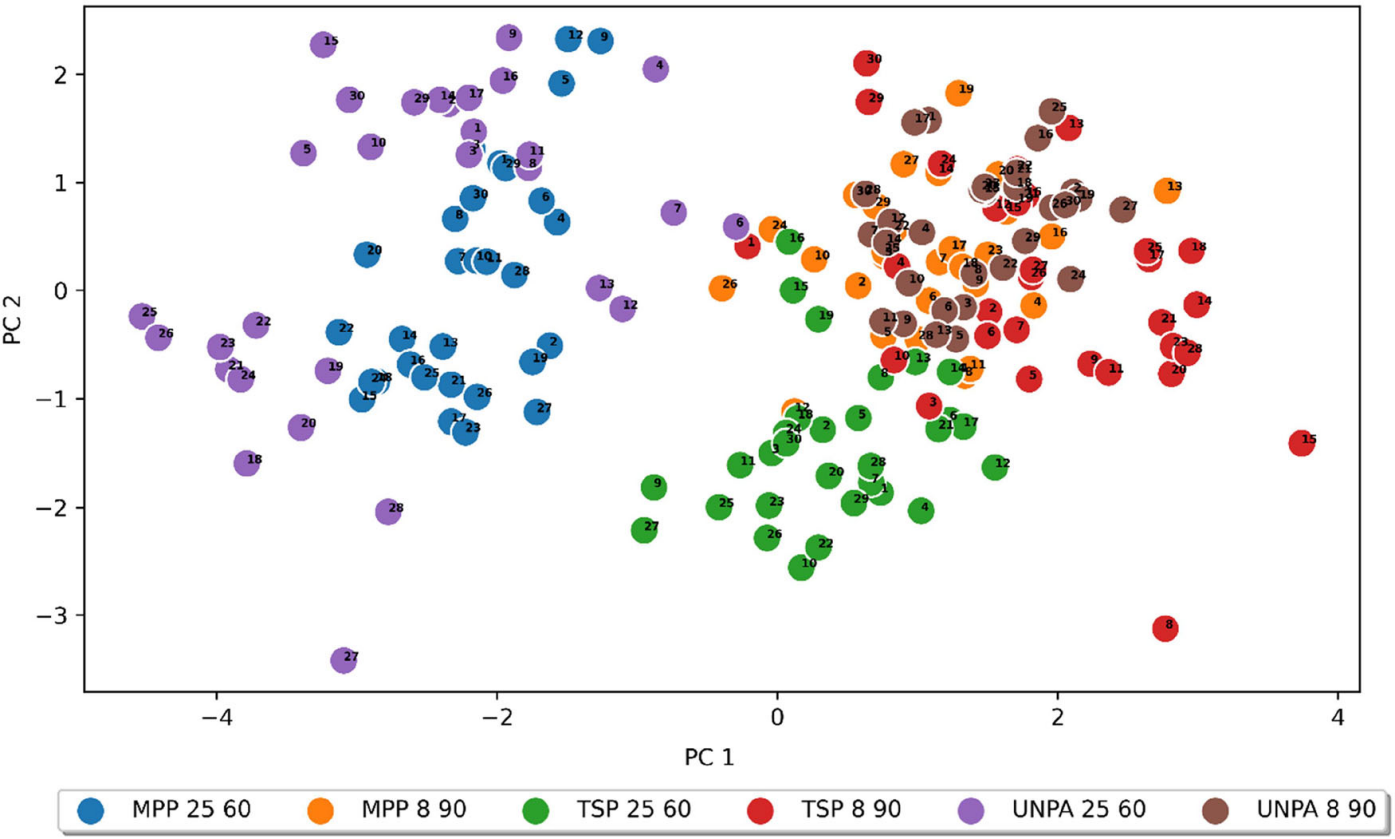
Factorial coordinates of the samples analyzed in the PCA analysis. (The number at the points indicates the number of repetitions.) MPP, macro‐perforated packaging; TSP, thermo‐sealable packaging; UNPA, unpackaged.

Figure 6 illustrates the distribution of points resulting from the PCA analysis in the system of the first two PCs, considering the storage conditions and packaging methods of the peppers. In the presented graph, all samples stored at 8°C are grouped closely together, suggesting that, at lower temperatures, the packaging method has little effect on the quality of the peppers. In the case of samples stored at 25°C, two of them (unpacked (UNPA 25 60) and packed in macro‐perforated foil (MPP 25 60)) overlap, although to a lesser extent than at lower temperatures. This indicates that storing in macro‐perforated foil has a rather negligible impact compared to storage without packaging. Storage in thermo‐sealable foil at a higher temperature (TSP 25 60) leads to a clear separation of points associated with peppers stored under these conditions. This separation is evident both at the level of the first PC, which is mainly correlated with losses in mass and dimensions (length, width, and thickness), and at the level of the second PC, which is primarily correlated with h* and ΔE*. The group of points representing samples packed in thermo‐sealable foil and stored at 25°C (TSP 25 60) is visibly shifted towards the samples stored at a lower temperature, indicating that the thermo‐sealable foil provides good protection for the raw material.

**FIGURE 7:**
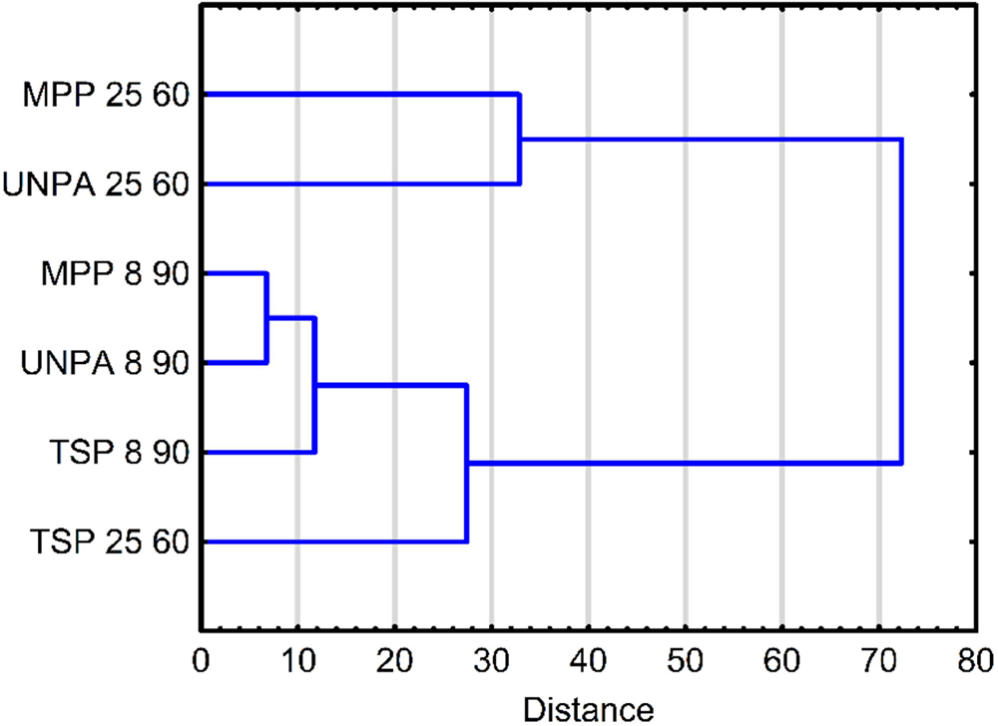
Dendrogram showing the segmentation of samples based on the average values of nine quality parameters of tested peppers samples without packaging and those packaged in macro‐perforated foil and thermo‐sealable foil stored at 8°C and 25°C. MPP, macro‐perforated packaging; TSP, thermo‐sealable packaging; UNPA, unpackaged.

HCA, focused on the segmentation of samples based on the similarities in the average values of the nine quality parameters, led to conclusions similar to those obtained from the PCA. The dendrogram shows that the samples without packaging and those packaged in macro‐perforated foil stored at high temperatures (UNPA 25 60 and MPP 25 60) were the first to separate, indicating that both storage methods have a similar effect on the tested pepper quality (Figure 7). Like the PCA, the dendrogram also shows that storing peppers in thermo‐sealable foil at high temperatures provides effective protection. These samples (TSP 25 60) are clearly separated from the other high‐temperature stored samples and are grouped in a branch with those stored at low temperatures.

However, at the next level of division, these samples are further distinguished from the low‐temperature group, suggesting that although thermo‐sealable foil improves preservation, it does not offer the same level of protection as refrigeration. Among the low‐temperature samples, those without packaging (UNPA 8 90) and in MPP (MPP 8 90) form a distinct group that separates from the samples stored in thermo‐sealable foil under refrigerated conditions (TSP 8 90). This suggests that packaging in thermo‐sealable foil and storage at 8°C with 90% humidity was the most effective among the tested treatments for maintaining pepper quality. This was also reflected in the lowest mass and length loss observed in these samples.

The potential of machine learning approaches in food analysis has been highlighted in previous studies (Moreira et al. 2019). Therefore, in the further considerations of this study, Kohonen networks were also applied for the classification of pepper samples based on their quality parameters. In contrast to previously used analyses, Kohonen networks provide nonlinear dimensionality reduction and flexible data clustering, making them a valuable alternative for better representing the complex structure and dependencies in the data, in addition to offering an intuitive visualization of clusters on a topological map (Kohonen 1982). These networks operate by tuning nodes to recognize specific input patterns or pattern categories, enabling them to effectively cluster high‐dimensional data. The study examined different two‐layer topologies of KNNs, where the input layer consisted of nine neurons feeding input values to the network. To develop an effective classification model, the size of the output layer, structured as a two‐dimensional grid (i.e., the number of neurons acting as nodes), and the network parameters were adjusted during the optimization process. Finally, the KNN, containing 25 neurons (5 × 5) in the output layer, characterized by the lowest QEs (defined as the average Euclidean distance between each input vector and its weight vector for the BMU), was selected to classify the examined pepper samples and rank the storage methods and parameters based on their effectiveness (Table 5).

**TABLE 5:** The quantization errors (QE) of the Kohonen neural network (KNN) model accuracy evaluation.

KNN model	Quantization erroe		
	Training dataset	Test dataset	Validation dataset
SOFM 9–25	0.0834	0.1294	0.1344

**FIGURE 8:**
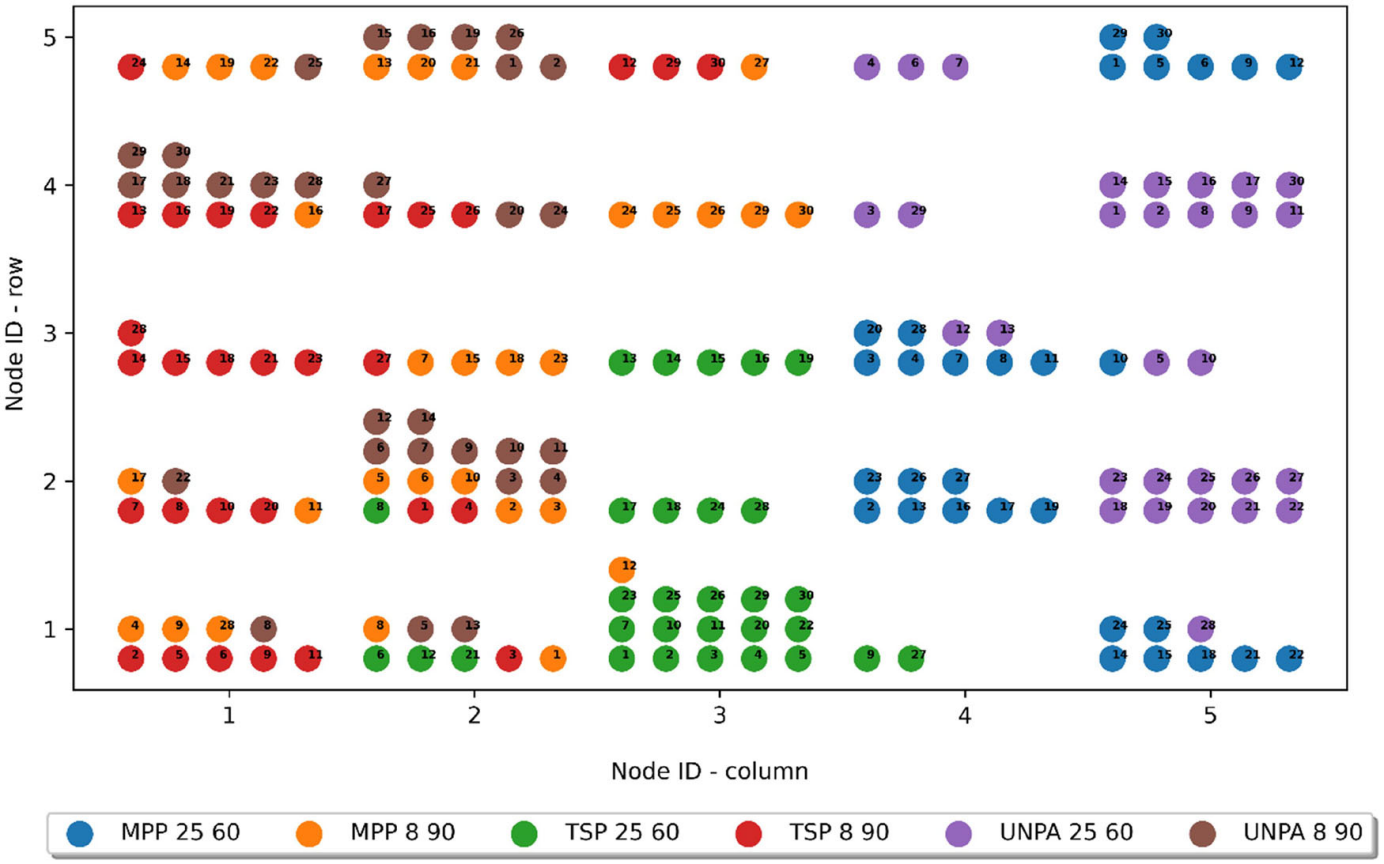
Distribution profiles of the analyzed samples of red pepper obtained by the architecture of the Kohonen neural network with 5 × 5 nodes in grid of output layer. MPP, macro‐perforated packaging; TSP, thermo‐sealable packaging; UNPA, unpackaged.

Figure 8 shows the distribution profiles of the analyzed pepper samples obtained from the designed KNN model. The selected KNN produced results similar to those of PCA and the dendrogram. All samples stored at lower temperatures (UNPA 8 90, TSP 8 90, MPP 8 90) formed a distinct cluster on the left side of the topological map, with a slightly smaller spread toward the bottom. Samples stored at higher temperatures without packaging or in macro‐perforated foil (UNPA 25 60 and MPP 25 60) constituted a second group on the right side of the network response map, also exhibiting a slightly reduced extent at the bottom. Meanwhile, the sample stored in thermo‐sealable foil at 25°C (TSP 25 60) formed a clearly separate cluster in the lower central region of the topological map, indicating a marked difference in the quality of these peppers compared to the other identified groups. This conclusion aligns with the results of PCA and HCA analyses, confirming that pepper storage at higher temperatures in thermo‐sealable foil is more effective than leaving them unpacked or packing them in macro‐perforated foil. However, although this method improves preservation, its effectiveness remains slightly inferior to that of storing peppers at low temperatures using any of the three packaging methods.

The results of the classification conducted using KNNs demonstrated their usefulness in recognizing the optimal pepper packaging method and storage conditions based on quality attributes. The effectiveness of SOMs in selecting key volatile compounds was also noted by High et al. (2021) during the maturation process of blue cheese.

It is worth emphasizing that the use of different, complementary data analysis techniques leads to synergy in data interpretation and classification. On one hand, a substantive interpretation of PCs allows for a better understanding of the nature of the data and helps identify the main directions of variance in the original dataset. On the other hand, although KNNs may be less straightforward in their interpretation, as they map data in a less transparent manner, they are more suitable for accurately modeling nonlinear patterns. In turn, a dendrogram illustrates the hierarchical relationships between objects, showing how different groups of data relate to each other at varying levels of similarity.

The comparison of findings from KNN analyses showed that the outcomes are consistent with the patterns observed in PCA and HCA. The KNN model, similar to the other two methods, identified distinct groupings of samples stored at 8°C, regardless of the packaging method, whereas unpackaged samples and those packed in macro‐perforated foil stored at 25°C formed another group. Furthermore, all conducted analyses highlighted the distinct characteristics of red peppers stored at 25°C in thermo‐sealable foil, suggesting that this packaging method, when used at higher storage temperatures, is the most effective among those tested for minimizing quality deterioration.

Satisfactory results in data interpretation through the integration of multiple multivariate analysis techniques have been confirmed in other studies. In one recent investigation, the combined application of PCA and KNN proved to be an effective approach for classifying honey samples (Koraqi et al. 2025). The use of both techniques allowed the authors to perform a deeper analysis of the relationships between physicochemical quality attributes, enabling precise differentiation of honey according to botanical origin. In another study, the combination of the NIR technique with PCA and KNNs revealed intricate interdependencies between NIR spectral variables and chocolate composition, particularly in relation to cocoa content (Lima et al. 2025). Similarly, Barros et al. (2021), comparing PCA, HCA, and Kohonen SOMs in the classification of edible seeds based on phenolic content and antioxidant activity, concluded that KNNs provided the most straightforward multivariate classification.

In this study, the consistency of findings across PCA, KNN, and HCA confirms the reliability of the analyses and demonstrates that the combined use of PCA, HCA, and KNN provides valuable insights into the interaction between red pepper quality attributes and various packaging and storage conditions. Additionally, it helps identify methods and conditions that are highly beneficial in practice for maintaining the quality of stored fresh red peppers.

## 4 Conclusions

The study incorporates statistical and machine learning techniques (PCA, HCA, and KNN) to investigate the relationship between red pepper quality parameters and packaging methods combined with various storage conditions. The conclusions resulting from the individual analyses were consistent providing complementary insights and strengthening the robustness of the findings. This multi‐method approach enabled the mitigation of the individual limitations of these methods, leading to their complementary effect in data pattern recognition and analysis of the impact of packaging and storage conditions on red pepper freshness, allowing the identification of its optimal packaging method and storage conditions. The obtained results can serve as useful practical guidelines for producers and storekeepers involved in the process of preserving the highest quality fresh peppers.

## Author Contributions

**Eugénio da Piedade Edmundo Sitoe**: conceptualization, investigation, funding acquisition, writing – original draft, methodology, validation, visualization, writing – review and editing, software, formal analysis, project administration, resources, supervision, data curation. **Clara Mariana Gonçalves Lima**: supervision, data curation, formal analysis, visualization, validation, writing – review and editing, writing – original draft. **Jolanta Wawrzyniak**: data curation, supervision, formal analysis, software, writing – review and editing, visualization, validation, methodology, writing – original draft. **Matheus da Silva Mourão**: investigation, writing – original draft, methodology, visualization, writing – review and editing, formal analysis, supervision.

## Conflicts of Interest

The authors declare no conflicts of interest.

## Data Availability

The data supporting the results of this article are available from the authors upon reasonable request.
